# The therapeutic potential of bacteriocins as protein antibiotics

**DOI:** 10.1042/ETLS20160016

**Published:** 2017-04-21

**Authors:** Hannah M. Behrens, Anne Six, Daniel Walker, Colin Kleanthous

**Affiliations:** 1 Department of Biochemistry, University of Oxford, South Parks Road, Oxford OX1 3QU, U.K.; 2 Institute of Infection, Immunity and Inflammation, College of Medical, Veterinary and Life Sciences, University of Glasgow, Glasgow G12 8QQ, U.K.

**Keywords:** antibiotics, bacteriocins, colicin, infection, pyocin

## Abstract

The growing incidence of antibiotic-resistant Gram-negative bacterial infections poses a serious threat to public health. Molecules that have yet to be exploited as antibiotics are potent protein toxins called bacteriocins that are produced by Gram-negative bacteria during competition for ecological niches. This review discusses the state of the art regarding the use for therapeutic purposes of two types of Gram-negative bacteriocins: colicin-like bacteriocins (CLBs) and tailocins. In addition to *in vitro* data, the potency of eight identified CLBs or tailocins has been demonstrated in diverse animal models of infection with no adverse effects for the host. Although the characteristics of bacteriocins will need further study, results obtained thus far regarding their *in vivo* potency, immunogenicity and low levels of resistance are encouraging. This leads the way for the development of novel treatments using bacteriocins as protein antibiotics.

## Introduction

Antibiotic resistance has increased at an alarming rate worldwide, yet at the same time the discovery of new antibiotics is stalling. Of particular concern are infections caused by Gram-negative bacteria, which possess a highly impermeable outer membrane that limits the entry of multiple classes of antibiotics, leaving limited therapeutic options that are increasingly less efficacious as resistance spreads [1–4]. Foremost among the Gram-negative pathogens are *Pseudomonas aeruginosa*, *Escherichia coli*, *Klebsiella pneumoniae* and *Acinetobacter baumannii*, all of which pose serious threats to global healthcare and patient safety; in 2008, US intensive care units reported 17, 13, 13 and 74%, respectively, of clinical isolates of these species as multidrug-resistant [5]. Among these pathogens, *E. coli* and *K. pneumoniae* are well-known producers of extended-spectrum β-lactamases and carbapenemases that render producing bacteria resistant to β-lactams including cephalosporins and carbapenems, which are considered to be antibiotics of last resort [2,6,7]. In addition, chronic biofilm-mediated infections, such as *P. aeruginosa* lung infections in cystic fibrosis patients and *E. coli* urinary tract infections, are relatively poorly treated with existing antibiotics [8–10]. Consequently, treatment options for infections caused by Gram-negative bacteria are limited and patient outcome is increasingly poor [8–10].

Bacteriocins offer a potential alternative therapeutic strategy to treat both multidrug-resistant and chronic bacterial infections. These antimicrobial peptides or proteins are diverse and widespread among Gram-positive and Gram-negative bacteria [11]. It is becoming evident that they are linked to pathogenesis as they play a role in displacing bacterial flora, enhancing colonization and therefore infection ([11–13] and Sharp et al. unpublished data). In fact, protein bacteriocin-encoding genes can be found in the genomes of most Gram-negative pathogens, including *P. aeruginosa*, *E. coli* and *K. pneumoniae* ([14] and Sharp et al. unpublished data). Importantly, they are highly specific antibacterials that kill only bacteria closely related to the producer and are deployed during the fight for resources with competitor strains [14–16]. This specificity makes them attractive as therapeutics as they offer a more targeted approach. Indeed, one major issue with conventional antibiotics is the dysbiosis induced by broad-range killing of bacteria [17]. While the narrow killing spectrum of bacteriocins means that the bacteria responsible for the infection have to be identified prior to treatment, it gives the advantage of being able to specifically target one species, or even one strain of bacteria, leaving the normal healthy microflora intact [17]. The narrow killing spectrum also reduces the selective pressure for resistance on bystander microorganisms [18].

Gram-negative bacteriocins can be classified into three groups: peptide bacteriocins, which have been reviewed elsewhere [19,20], colicin-like bacteriocins (CLBs) and tailocins. The potential of the latter two, CLBs and tailocins, to treat bacterial infections is reviewed here. Previous reviews have discussed Gram-positive bacteriocins as antibiotics [11,21,22] and the *in vitro* potential of bacteriocins against Gram-negative bacteria [11,23–25]. Our aim is to focus on the potential ability of CLBs and tailocins to treat Gram-negative infections and give an exhaustive report of the results available from *in vivo* models of infection to date. We will not discuss data from probiotic applications of bacteriocins as the role of bacteriocins in probiotics is complex and only beginning to be understood [26], and although recent progress has been made in understanding the role of peptide bacteriocins in *E. coli*, equivalent investigations for CLBs and tailocins are still lacking [27].

*In vitro* studies have demonstrated the activity of bacteriocins against both planktonic and biofilm-embedded bacteria [28–31] and, interestingly, Rendueles et al*.* demonstrated that colicin R preferentially targets bacteria growing in the biofilm state. As a result, there is renewed interest in investigating the efficacy of bacteriocins in animal models. The use of animal models of infection to test antibacterial compounds is a long-established practice and acknowledged as a useful tool in predicting if translation into the treatment of human infections is likely to be successful. While there is no such thing as a standard animal model, new models have been developed to more closely resemble specific human infections (acute or chronic infections, sepsis and meningitis), giving improved insights into host–pathogen interactions during antibiotic treatment (reviewed in ref. [32] for *P. aeruginosa*). Since their discovery, bacteriocins have been tested in a variety of animal models, with a range of modes of administration and dosing regimens. Here, we summarize the results of these investigations and discuss factors that will ultimately determine the success of bacteriocins as therapeutic agents. Although further research is required, bacteriocins have key properties such as high potency *in vivo* and exquisite specificity that suggest they may be excellent candidates for further therapeutic development.

## *In vivo* activity of bacteriocins

### Colicin-like bacteriocins

Colicins are 40–70 kDa toxins that specifically target *E. coli*. Given the fact that these molecules were the first to be discovered and have been the best studied to date [33], we refer to similar molecules produced by other Gram-negative bacteria as CLBs. CLBs target strains closely related to the producing strain, typically of the same species, and have species-specific names: colicins from *E. coli*, klebicins from *Klebsiella* and pesticins from *Yersinia pestis*. *Pseudomonas* CLBs are named S-type pyocins (named after *P. pyocyanea*) [34] and are produced by >70% of strains [35]. CLBs are protease-susceptible, modular proteins with domains involved in receptor binding, translocation and cytotoxicity.

**Figure 1.**
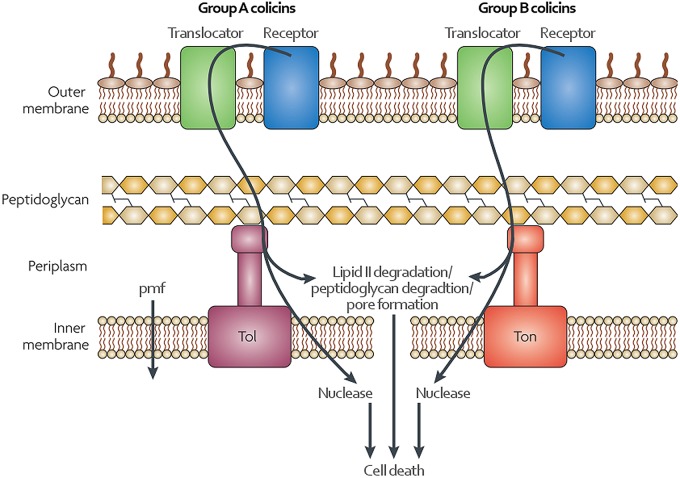
Import of colicin-like bacteriocins. Specificity of CLBs is mediated by binding a receptor in the outer membrane of a target cell. An adjacent translocator will then mediate transport into the periplasm. Energy for this transport is provided by either the Tol system for group A CLBs or by the Ton system for group B CLBs. Pore-forming colicin-like bacteriocins then insert into the inner membrane to disrupt the proton motive force (pmf) while nucleases translocate into the cytoplasm. Adapted from ref. [36]**.**

Using their receptor-binding domain, CLBs bind to bacterial outer membrane proteins before translocation through either the Ton or Tol systems of Gram-negative bacteria [36]. Many CLBs exploit TonB-dependent transporters to enter the cell, the physiological role of which is to import siderophores or vitamins. Although less well understood, the Tol system is also required for the entry of some CLBs. Several CLBs, such as colicin N and pyocin L1, have also been shown to interact with lipopolysaccharides (LPSs) on the cell surface [37,38]. The cytotoxic domains of CLBs are commonly pore-forming ionophores or nucleases, but some also have lipid II- or peptidoglycan-degrading activity (Figure 1) [39]. For the producing organism to be protected from its own bacteriocin, it produces an immunity protein that, at least in the case of the nuclease CLBs, binds the cytotoxic domain and neutralizes its activity [15].

Although data are currently limited, the efficacy of both the colicins and S-type pyocins has been tested *in vivo*. Indeed, a previous study showed that colicin E1 in pig feed can prevent diarrhoea and increase growth and feed efficiency; however, the dose was not sufficient to completely eradicate the targeted pathogenic strain of *E. coli* [40]. Pyocin S2 was shown to protect against a lethal *P. aeruginosa* infection in the greater wax moth *Galleria mellonella* larvae, a model of *P. aeruginosa* infections [41], and in murine lung infections when given 1 h post-infection [42]. The latter effect was also observed with pyocins AP41, S5 and L1, with pyocin S5 being particularly potent. Indeed, pyocin S5 was at least two orders of magnitude more potent than tobramycin in this model (Table 1) [42]. Similarly, all four pyocins were protective when given 6 h before infection. Interestingly, none of the bacteria isolated after pyocin treatment were resistant to the pyocins used, one isolate displaying increased tolerance to pyocin AP41. However, infection with this pyocin AP41-tolerant strain could still be controlled with this pyocin *in vivo*. Combinations of pyocins were also tested *in vivo* and were found to reduce colony forming units counts further, although not significantly [42].

**Table 1:** Effectiveness of protein antibiotics in *in vivo* challenge models

Model host	Protein	Treatment route	Challenge organism	Challenge route	Prophylactic (P) or therapeutic (T)?*	Effective?	
	**Colicin-like bacteriocins**						
Wax moth *G. mellonella* larvae	Pyocin S2	Injection	*P. aeruguinosa*	Injection	T	Yes	[29]
Mouse	Pyocin S2	IN	*P. aeruguinosa*	IN	P	Yes	[42]
	Pyocin AP41					Yes	
	Pyocin S5					Yes	
	Pyocin L1					Yes	
	Pyocin S2				T	Yes	[42]
	Pyocin AP41					Yes	
	Pyocin S5					Yes	
	Pyocin L1					Yes	
Pig	Colicin E1	Oral	*E. coli*	Oral	P	Yes	[40]
	**Tailocins**						
Mouse	Pyocin R2	IP	*P. aeruginosa*	IP	T	Yes	[43]
		IV			T	Yes	
Mouse	Enterocoliticin	Oral	*Yersinia enterocolitica*	Oral	T	Yes	[49]
	**Engineered tailocins**						
Rabbit	AvR2-V10.3	Orogastrically	*E. coli*	Orogastrically	T	Yes	[63]
	**Unknown and unidentified bacteriocins**						
Mouse	Unknown pyocin(s) P10	IP	*P. aeruginosa*	IP	P	Yes	[52]
		IP		IP	T	No	
Chick embryo	Unknown pyocin	Allantoic cavity	*P. aeruginosa*	Allantoic cavity	P	Yes	[50]
Mouse	Pyocin H108 (S-type)	IP	*P. aeruginosa*	IP	P	No	[54]
					T	No	
Mouse	Colicin ‘wash’	‘Bladder wash’	?	‘Established UTI’	T	Yes	[53]
Mouse	Unknown pyocin 78-C2	IV	*P. aeruginosa*	IV	P	Yes	[51]
		IP				No	
Mouse	Pyocin 1577 (R-type)	IP	*P. aeruginosa*	IP	P	Yes	[54]
					T	No	
	Pyocin 5882 (F-type)				P	Yes	
					T	No	
	Pyocin 1577 (R-type)	Topical		Topical on burn	T	No	
		IV			T	No	
	Pyocin 5882 (F-type)	Topical		Topical on burn	T	Minimal	

Detailed description of studies with known and identified bacteriocins can be found in the text. Abbreviations: IV, intravenous; IP, intraperitoneal; IN, intranasal; UTI, urinary tract infection. *Bacteriocin given at the same time as the challenge is considered prophylactic.

### Tailocins

**Figure 2.**
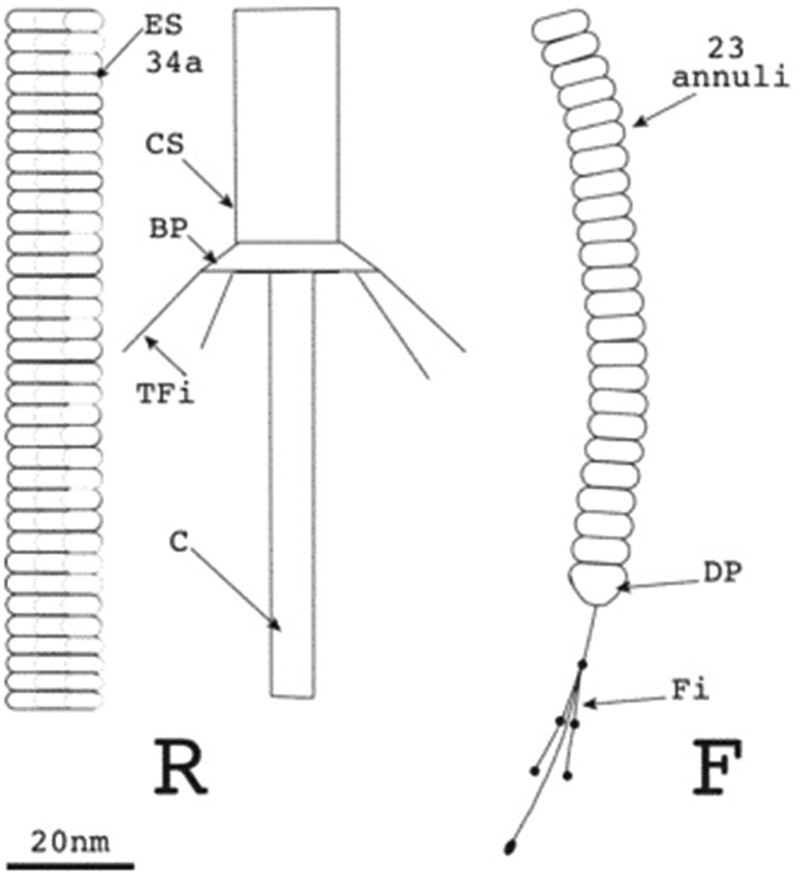
R-type tailocins consist of an inner core (C) and sheath, which can be contracted (CS) or extended (ES). Upon binding a target cell with tail fibres (TFi), attached to the sheath via the base plate (BP), the sheath contracts and inserts the core into the inner membrane of a target cell, consequently depleting the proton motive force. F-type tailocins consist of a flexible rod, made from 23 annuli, to which fibres (Fi) with some globular structures are attached via a distal part (DP). Taken from ref. [46].

Tailocins, also called high molecular mass bacteriocins, resemble the tail structures of bacteriophages from the Myoviridae and Siphoviridae families [39]. Although they are thought to be derived from phages, they are not simply defective phages, but are adapted specifically to their function as bacteriocins [43]. Tailocin gene clusters are found in the genomes of many Gram-negative and Gram-positive bacteria [44,45]. The best studied tailocins are the R- and F-type pyocins of *P. aeruginosa.* R-type pyocins are morphologically and genetically similar to the contractile tail of P2-like temperate enterophages [46]. They consist of a core and sheath, and are contractile (Figure 2). F-type pyocins resemble λ phage, have no sheath and are flexible (Figure 2) [39]. Tailocin specificity is modulated by tail fibres, which have been demonstrated to bind to LPSs, with different residues involved depending on the tailocin [47,48]. Upon contact with the receptor, the tailocin undergoes conformational changes that result in lethal membrane damage, detected as the depletion of the proton motive force. As opposed to CLBs, tailocins are not protease-susceptible and no immunity proteins have been described [39]. Very little is known about the mechanisms of resistance to tailocins; however, it is suggested to be mediated by an alteration of cell surface receptors recognized by the tailocin [47].

Studies of tailocins in animal models have shown that, like CLBs, they show strong efficacy against Gram-negative infections. Scholl and Martin [43] conducted a thorough study of pyocin R2 efficacy in a murine peritonitis model. They determined the time window after infection in which treatment is possible and the dose necessary for adequate treatment. Protection via immune system stimulation was ruled out and pyocin R2 was found to be an effective therapeutic up to 4 h after infection in an aggressive infection model. Their results suggest that higher or repeated doses would allow for later treatment. The treatment of a second infection was less effective due to protective antibodies that had formed. They also found that the effective dose of pyocin R2 to treat the intraperitoneal (IP) challenge was lower when delivered through IP injection than through intravenous (IV) injection, suggesting that site-specific delivery of this pyocin can improve its efficacy (Table 1) [43].

Enterocoliticin, an R-type tailocin from *Yersinia enterocolitica*, was found to be effective for the treatment of mice orally infected with *Y. enterocolitica* when given orally [49]. The tailocin was only found to be active *in vivo* for 5 h; however, doses were given up to 24 h apart. Taking this into consideration, only those mice that received enterocoliticin 1 h after infection and were analyzed 5 h later can inform on the effect of the tailocin, and these mice did indeed show significantly reduced bacterial numbers (Table 1) [49].

Unfortunately, many CLBs and tailocins that were shown to be active *in vivo* [50–55] cannot be identified today, due to incoherent naming and lack of identification of the active molecules (Table 1).

### Engineered bacteriocins

An important aspect of bacteriocin structure and function is their organization into domains that can be engineered to generate chimeric bacteriocins. Indeed, domains from different CLBs can be combined to customize their features. These include the target specificity via change of the receptor recognition domain, the immunity profile via change of immunogenic domains, mode of action via change of the cytotoxic domain and stability via change of structure, length or amino acid composition.

A combination of receptor binding and translocation domains of the CLB pesticin and bacteriophage T4 lysozyme was shown to be active against *Y. pestis* in mice [56]. The cytotoxic domain of CLBs can be combined with other pathogen-targeting proteins or domains, such as phage proteins [57], pheromones [58,59], antibodies or parts thereof, or ligands [60]. Combinations of the colicin Ia killing domain with pheromones, PMC-SA [58] against Gram-positive *Staphylococcus aureus* and PMC-EF [61] against Gram-positive *Enterococcus faecalis*, have been tested *in vivo* and were found to be effective.

Tailocin chimeras can be created by combining tailocins with parts of other tailocins or phages [62,63]. As the specificity of the tailocin is mediated by tail fibres, replacing them can enable the complex to recognize new targets [46]. AvR2-V10.3, an R-type pyocin with tail fibres engineered to target *E. coli* O157:H7, was shown to be effective in a diarrhoea model in rabbits, even when given after the onset of diarrhoea [63].

## Bacteriocins as protein antibiotics

While data on the use of bacteriocins as protein antibiotics obtained in the last few years from *in vivo* experiments are encouraging, there are key questions related to their suitability as therapeutics that remain to be addressed. First, only preliminary data on the effect of bacteriocins on the host in terms of toxicity or immune response are available. However, the limited data that are available in the majority of cases report no adverse or toxic effects against the host organism (Table 2). There is one exception to this: Bird and Grieble [50] observed 11% mortality in pyocin-treated chick embryos, while in the control group 6% mortality occurred from injection alone, although it is not clear whether this pyocin preparation was free of endotoxin. In addition, more research is required on dosing regimens and in particular the timing of bacteriocin administration pre- or post-infection. To address this, testing in well-defined infection models and detailed pharmacokinetic studies are required.

**Table 2:** Adverse effects of protein antibiotics *in vivo*

Model host	Protein	Treatment route	Adverse effect	
**Colicin-like bacteriocins**				
Pig	Colicin E1	Oral	None	[40]
*G. mellonella* caterpillar	Pyocin S2	Injection	None	[41]
Mouse	Pyocin S2	IN	None	[42]
	Pyocin AP41		None	
	Pyocin S5		None	
	Pyocin L1		None	
**Tailocins**				
–	–	–	–	–
**Unknown and unidentified bacteriocins**				
Mouse	Unknown pyocin 78-C2	IV	None	[51]
		IP	None	
Mouse	Unknown pyocin(s) P10	IP	None	[52]
Chick embryo	Unknown pyocin	Allantoic cavity	11% mortality after pyocin treatment and 6% mortality in controls	[50]
Mouse	Pyocin H108 (S-type)	IP	None	[54]
Mouse	Pyocin 1577 (R-type)	IP	None	[54]
	Pyocin 5882 (F-type)		None	
	Pyocin 1577 (R-type)	Topical	None	
	Pyocin 5882 (F-type)		None	
Mouse	Unknown pyocin (tailocin)	IP	None	[55]
Rabbit		SC	None	

Abbreviations: IV, intravenous; IP, intraperitoneal; IN, intranasal; SC, subcutaneous.

What about immunogenicity? One might assume that bacteriocins and phage proteins are not immunogenic as they have been employed in bacterial and viral warfare within our bodies for millennia. Nonetheless, some groups have found low levels of antibodies against these proteins [42,43]. Repeated exposure to high doses of pyocin S5 via the IP route in mice did not lead to the development of pyocin S5-specific IgA or IgG, whereas exposure via the intranasal route led to low levels of S5-specific IgG, which did not interfere with pyocin S5 treatment of subsequent infection [42]. Conversely, another study demonstrated that a single IV exposure of pyocin R2 to mice induced the production of neutralizing antibodies, consequently making a second treatment ineffective in 90% of mice. Four of five mice that died in the second treatment round had formed neutralizing antibodies [43]. Overall, immunogenicity has barely been investigated and more research is required.

Comparison of the potency of bacteriocins with that of conventional antibiotics has elicited encouraging results. Indeed, pyocin S5 is at least 100-fold more potent than tobramycin in a murine lung infection model, making pyocin S5 a promising replacement for traditional antibiotics to target *P. aeruginosa* [42]. The same study demonstrated that pyocins are stable and active *in vivo* for at least 24 h [42]. However, most protein antibiotic candidates are not as stable as traditional antibiotics and may need to be applied in higher doses and with shorter dosing intervals between repeated doses. While this may be perceived as a disadvantage, low stability can also reduce whole-body and environmental exposure to the antibiotic, which may minimize the selective pressure for the development of resistance [64].

Finally, at the origin of the antibiotic crisis, we are currently facing is the emergence of resistance to conventional antibiotics. While emergence of resistance to conventional antibiotics is widely documented, little is known about how resistance to bacteriocins might arise and evolve *in vivo*. Several studies have shown resistance to bacteriocins in environmental settings and that resistance to bacteriocins can occur through modification of cell surface receptors *in vitro*. Recent results suggest that mechanisms of resistance are dependent on environmental factors and that resistance may be reduced *in vivo* [17,65]. It is interesting to note that while resistance is possible, many bacteria remain sensitive to a certain level to the bacteriocin, suggesting that complete resistance is hard to evolve or comes at a great cost for the bacteria [66].

Immunity, as opposed to resistance, is mediated by immunity proteins that specifically bind CLBs. The natural immunity of bacteriocin-producing strains could prove to be a drawback to the use of bacteriocins as protein antibiotics. However, it has recently been shown that immunity, too, comes at a great cost, as immune strains are outcompeted by non-immune strains under non-selective conditions [67]. Moreover, while some bacteriocins share killing mechanisms or have identical cytotoxic domains that could lead to cross-immunity between bacterial strains, it is to be noted that many different killing mechanisms exist. These varied mechanisms limit the emergence of cross-resistance and immunity among protein antibiotics. It is also interesting to note that most bacterial strains do not express CLBs and among those that do, the majority produce only one CLB (Sharp et al. unpublished data). This suggests that most strains will either be susceptible to bacteriocins or be immune to a limited number of them. Therefore, it is expected that the use of a cocktail of bacteriocins will be able to circumvent individual immunity. Interestingly, in rare cases, *in vitro* susceptibility of strains to protein antibiotics did not correspond to *in vivo* susceptibility [51,68].

## Conclusions

Commercial interest in bacteriocins comes from many sides. Farming industries, such as the poultry industry [21,69] and aquaculture industry [70], look for more efficient ways to treat their stock. Interest in human applications comes from scientists and companies that realize the global health and economic potential of bacteriocin therapeutics. As early as 1986, a potential application of colicin E1 was patented [71]. Many researchers have since patented applications of CLBs to treat infections [41,60,68,72,73]. Two patents focus on the delivery of CLBs to specific organs [68,74]. Scientists at Avid-Biotics Corporation have been engineering tailocin tail fibres for over a decade, resulting in five patents [75–79] with either diagnostic or therapeutic aims.

Eventually, application of probiotic bacteria-producing bacteriocins rather than application of purified bacteriocins might be more cost-effective. Recently, production of bacteriocins in plants has been achieved at costs that may encourage commercialization [80]. However, interactions between pathogenic and probiotic species in the setting of an *in vivo* microbiome are not yet understood well enough to predict their success [27].

This review provides an overview of *in vivo* studies undertaken to date. However, the lack of systematic identification and naming of bacteriocins have rendered the results of *in vivo* studies, e.g. that of Rosamund Williams [54], useless for posterity, highlighting the need for a uniform nomenclature accessible and used by all. While databases on bacteriocins exist (BACTIBASE, BAGEL), they mostly focus on Gram-positive peptide-type bacteriocins, leaving a big gap to be filled for Gram-negative bacteria, which also use protein bacteriocins.

Recently, over 2000 protein bacteriocins were identified across a range of Gram-negative bacteria including the important pathogens *P. aeruginosa*, *E. coli* and *K. pneumoniae* (Sharp et al. unpublished data). Less than 100 of these have been studied *in vitro* [15] and only eight of them have been studied *in vivo*. More preclinical studies are required to better characterize bacteriocins, especially their immunogenicity and the emergence of resistance *in vivo*. However, the results of the reviewed *in vivo* experiments, as well as the use of bacteriocins against Gram-positive bacteria such as nisin as a food preservative and topical antibiotic in humans [81], are encouraging. They showcase the potential that might be hidden among the thousands of unstudied bacteriocins in terms of therapeutics and indicate promise for the use of bacteriocins as protein antibiotics.

## Summary

- Bacteriocins have been shown to be effective in a range of acute infection models and offer the potential for highly targeted antibiotic therapy.
- There is a lack of key data on issues such as the ability of bacteria to develop resistance to bacteriocins *in vivo* and the toxicity and immunogenicity of bacteriocins on repeat administration.
- High quality preclinical data that address these key questions are required to support the development of bacteriocins as therapeutic protein antibiotics.

## Abbreviations

CLBs: colicin-like bacteriocins
IP: intraperitoneal
IV: intravenous
LPSs: lipopolysaccharides
UTI: urinary tract infection
